# Pressure gradient prediction in aortic coarctation using a computational-fluid-dynamics model: validation against invasive pressure catheterization at rest and pharmacological stress

**DOI:** 10.1186/1532-429X-17-S1-Q78

**Published:** 2015-02-03

**Authors:** Julio A Sotelo, Israel Valverde, Philipp B Beerbaum, Gerald F Greil, Tobias Schaeffter, Reza Razavi, Daniel E Hurtado, Sergio Uribe, Carlos A Figueroa

**Affiliations:** 1 Biomedical Imaging Center, Pontificia Universidad Católica de Chile, Santiago, Chile; 2 Electrical Engineering, Pontificia Universidad Catolica de Chile, Santiago, Chile; 3 Pediatric Cardiology Unit, Hospital Virgen del Rocio, Seville, Spain; 4 Cardiovascular Pathology Unit, Institute of Biomedicine of Seville (IBIS), Seville, Spain; 5 Hannover Medical University, Hannover, Germany; 6 Division of Imaging Sciences and Biomedical Engineering, King's College London, London, UK; 7 Structural and Geotechnical Engineering, Pontificia Universidad Catolica de Chile, Santiago, Chile; 8 Radiology, School of Medicine, Pontificia Universidad Catolica de Chile, Santiago, Chile; 9 Surgery and Biomedical Engineering, University of Michigan, Ann Arbor, MI, USA

## Background

Aortic Coarctation (AoCo) accounts for 5-8% of the children with CHD. Even after successful early repair, life expectancy is still markedly reduced (80% at 50 years after surgery) compared to normal population due to long term complications (hypertension). Usually, invasive diagnostic catheter investigations are required to evaluate the pressure gradient across the aorta at rest, or unmask such a gradient by use of isoprenaline stress to mimic physical exercise. The application of image-based computational fluid dynamics (CFD) in patients with AoCo appears promising as an alternative non-invasive diagnostic tool, as it may allow the avoidance of cardiac catheterization to determine pressure gradients. The motivation of this research is to know if a MRI based CFD model can accurately predict the pressure gradient in patients with AoCo and therefore be incorporated in the clinical practice.

## Methods

**Figure 1:**
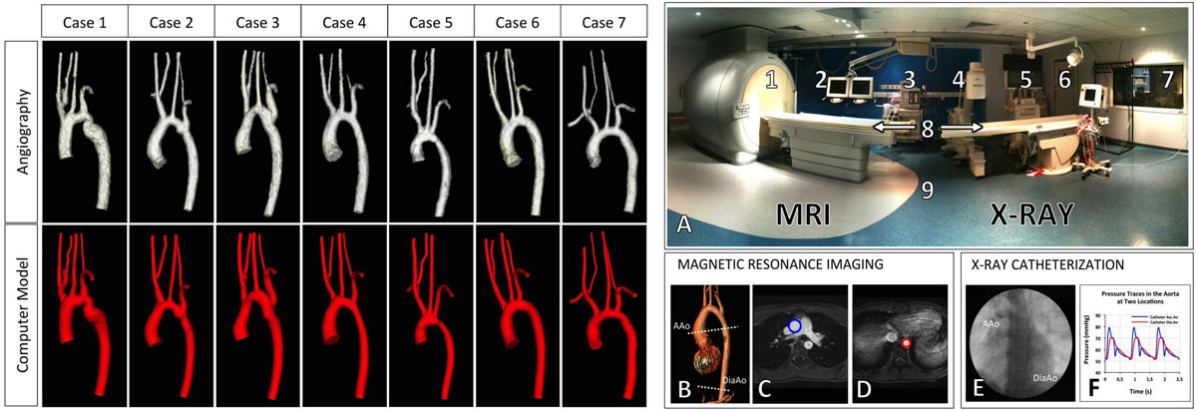
(Left) Magnetic resonance angiography (first row) and solid models used for the CFD simulation (second row). (Right) Combined MRI and X-Ray suite (XMR) for clinical investigations. The XMR suite (Panel A) consists of: 1. MRI scanner; 2. MRI monitor and controls; 3. Anesthetist equipment; 4. X-ray fluoroscopy unit; 5. X-ray monitoring; 6. Door to scrub room; 7. Control room; 8. Sliding tabletop; 9. Five Gauss color marking line. The MRI study included 3D CE angiography of the aorta (B), where the 2D PC-MRI image at level of the ascending aorta (AAo) are shown in (C) and the diaphragmatic aorta (DiaAo) in (D). The x-ray catheterization study included invasive pressure measurements at the level of the ascending and diaphragmatic aorta (E-F).

The study included 7 cases with aortic coarctation (mean ± standard deviation; age 19.4±4.6years, weight 71.9±17.1kg) (Fig. 1-Left), who had a previous combined MRI (3D CE-MRA, Fig. 1B, and 2D CINE-PC in the ascending and diaphragmatic aorta, Fig. 1C-D) and cardiac catheterization (Two femoral artery catheterization Fig. 1-Right) in a 1.5T Intera MRI scanner and BT Pulsera cardiac radiography unit, Philips, Best, Netherlands. The 3D CE-MRA data was used to create CFD models of the aorta (Fig. 1-Left) using SimVascular (simtk.org) and MeshSim (Simmetrix, Clifton Park, NY). The boundary condition (flows and stiffness distribution) of CFD was setting using the 2D PC-MRI and pressure data.

## Results

**Figure 2:**
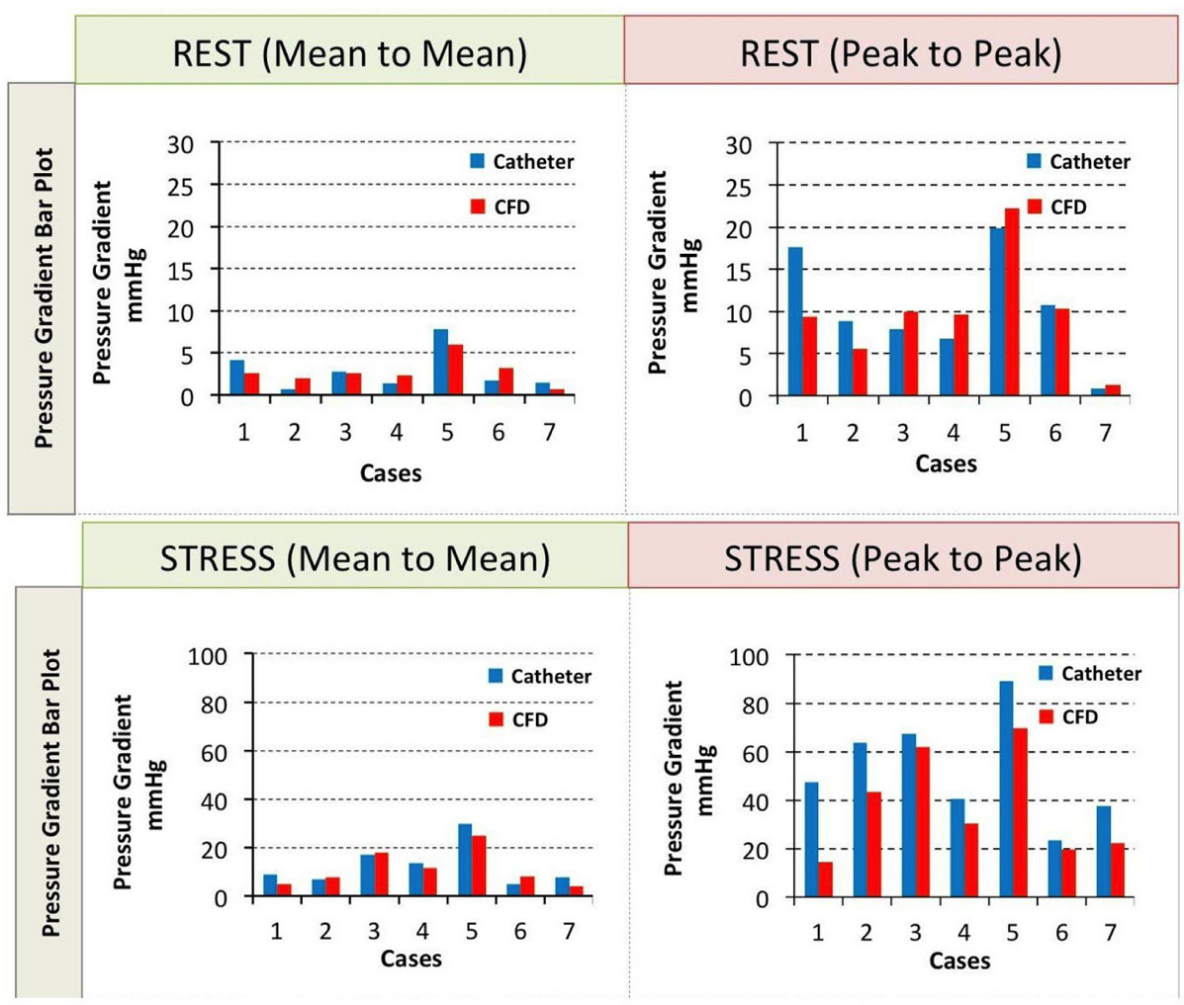
Simulation results, pressure gradient in rest condition (mean to mean and peak to peak) and stress condition (mean to mean and peak to peak), for all cases.

The pressure gradients obtained in REST were in good agreement with the ones obtained from catheterization Fig. 2. The mean-to-mean pressure gradient averaged between all cases was 2.85±2.47mmHg for the catheterization and 2.76±1.64mmHg for the simulation. The peak-to-peak pressure gradient, averaged between all cases was 10.36±6.54mmHg for the catheterization and 9.77±6.39mmHg for the simulation. In STRESS the mean-to-mean pressure gradient averaged between all cases was 12.59±8.61mmHg for the catheterization and 11.25±7.60mmHg for the simulation. The peak-to-peak pressure gradient, averaged between all cases of 52.71±22.11mmHg for the catheterization and 37.38±21.64mmHg for the simulation (Fig. 2).

## Conclusions

In conclusion, we can predict the non-invasive pressure gradient with a good agreement using CFD simulation and cardiovascular magnetic resonance imaging, with the purpose that in the future incorporate this process in the clinical practice.

## Funding

European Research Council under the European Union's Seventh Framework Programme (FP/2007-2013) / ERC Grant Agreement n. 307532, the United Kingdom Department of Health via the National Institute for Health Research (NIHR) comprehensive Biomedical Research Centre award to Guy's & St Thomas' NHS Foundation Trust in partnership with King's College London and King's College Hospital NHS Foundation Trust. FONDECYT #1141036 and #11121224. JS thanks CONICYT and Ministry of Education of Chile, with his higher education program, for scholarship for doctoral studies.

